# How do we reconcile the seemingly contradictory theories of Gestalt language processing and weak central coherence?

**DOI:** 10.3389/fpsyt.2025.1665247

**Published:** 2025-09-17

**Authors:** Anna M. Schwartz, Catherine L. Caldwell-Harris

**Affiliations:** ^1^ Department of Physical Therapy, Movement & Rehabilitation Science, Northeastern University, Boston, MA, United States; ^2^ Department of Psychology, Boston University, Boston, MA, United States

**Keywords:** autism, language acquisition, top-down and bottom-up approach, enhanced perceptual functioning, gestalt language processing, weak central coherence

## Abstract

A long-standing characterization of autistic cognition is variously referred to as detail-oriented, local processing bias, bottom-up processing, and weak central coherence. A related construct, enhanced perceptual functioning, is a mechanism that could account for autistics’ superiority at a variety of detail-oriented visual tasks. However, detail-focused and enhanced perceptual functioning appear the opposite of what is called Gestalt language processing. Observed in autistic language acquisition, whole phrases are produced at the beginning of language learning, rather than the expected smaller unit of single words. This idea has been enlarged to encompass Gestalt processing as a broad cognitive style, said to be characteristic of autistic processing in some cases or for some individuals. We explain and critique these diverse accounts, noting which aspects of them are controversial. We propose a spectrum of reconciliations, beginning with how both attention to detail and production of holistic speech can emerge from enhanced perceptual function. Inherent differences between auditory and visual processing allow holistic processing to be more easily observed in language than in vision. Autistic individuals may less easily learn what level and type of detail correspond to their culture’s normative expectations.

## 1 Introduction: local processing bias

Focusing on the trees and not the forest. Working on the details of a problem but ignoring the big picture. Over the last decades, these observations of autistic cognitive processing have been experimentally verified using diverse tasks. In the embedded figure task (1), a line drawing of a pie shape would be difficult to find if it was drawn by the spokes on the wheel of a baby carriage. Autistics were faster and more accurate than a neurotypical comparison group at identifying hidden shapes, presumably because of greater bottom-up processing, meaning focusing on details of the image, without conflicting top-down signals from the context surrounding the shape.

Another informative task was the local-global task, where observers viewed a large letter (such as G) composed of small images of a different letter (such as E). Observers are asked to respond as quickly as possible regarding which letter is being displayed. Autistics were more likely than neurotypical observers to respond with the small (or “local”) letter (2, 3). The frequency of local vs. global choices can then be used to quantify the relative predominance of local vs. global processing bias.

### 1.1 Weak central coherence

Happé and Frith (3) explained detail-oriented cognition using the phrase “weak central coherence,” where central coherence refers to integrating information coming from diverse aspects of domains. For example, in vision, integrating the distinct shape of leaves, branches, and a trunk helps identify the overall object, tree; integrating a scene with trees, hills and a sky would identify this as a landscape. Neuroanatomical evidence has also supported the idea that autistic brains have more connections in local areas, and fewer long-range connections (4–7). 8). Happé and Frith (3) argued that difficulty integrating information is a general characteristic of autistic functioning. Examples include integrating information when listening to sentence narratives (9–11), and difficulties making generalizations about categories, especially new categories (12, 13). Autistic individuals are less helped by context when taking memory tests and problem-solving (14). Happé and Frith (3) argued that difficulty with integrating information could also explain two key aspects of autism, restricted interests and social impairments. Specific, idiosyncratic interests are those that don’t depend on flexibly interrelated diverse types of conceptual information. Social skills deficits may emerge because the social domain requires a dynamic blend of facial emotion processing, gestures, and knowledge of social norms (see also 15).

### 1.2 Enhanced perceptual functioning

Mottron et al. (16) proposed that autistics have heightened sensitivity to sensory stimuli, particularly in the visual and auditory domains. This sensitivity leads to a detail-oriented cognitive style, where fine-grained information is prioritized for processing, at the expense of attention to global information(see review in 17). As part of reframing detail-orientation as a strength, Mottron et al. (16) showed that autistic outperformed neurotypicals on diverse tasks of block design, pattern recognition, and visual search. Children with ASD exhibited faster reaction times and no disruption in search efficiency on dynamic visual processing tasks (18). Those authors argued that the autistic advantage derives from enhanced perceptual ability to discriminate between targets and distractors, although the cause of this was not specified.

EPF reframed local processing bias as a strength, which also helped explain savants (19). EPF could be the mechanism by which some autistic individuals develop extreme talents such as calendrical calculation (20).

Mottron et al. (16) proposed that EPF is broadly about sensory processing, and not just visual processing. They reviewed that autistics have superior pitch discrimination, heightened ability to distinguish between different musical pitches, enhanced pure-tone detection. Relative to NTs, autistics have improved auditory pattern recognition. They excel at identifying and remembering complex auditory patterns and sequences, and have superior auditory stream segregation, meaning better ability to separate and process multiple simultaneous auditory streams. Many individuals with ASD report heightened sensitivity to certain sounds, which may be related to enhanced auditory perception. Autistic individuals reported during interviews being drawn to details in a visual scene and having enhanced perceptual processing, such as navigating through an environment with a detailed map-like mental representation (21).

### 1.3 Evolving views of the causes of enhanced perceptual functioning

It was older literature from the 1980s, 1990s, and early 2000s that used the terms detail-oriented, local processing bias, and weak central coherence. Another reframing came from researchers who argued that enhanced sensory functioning can be attributed, at least in part, to enhanced sensory capacity.

Remington et al. (22) designed tasks to stress visual processing capacity. Autistic adults and children had intact performance on a central attention task, but compared to an NT group, autistic adults had increased ability to process irrelevant peripheral information, even under high levels of perceptual load. Those authors concluded that autistics had superior visual processing ability compared to NTs (22, 23).

A proposed mechanism is increased auditory capacity, paralleling the increased visual attention advocated by Remington et al. (22, 23). To compare perceptual capacity in both autistics and NT groups, Remington and Fairnie (24) designed auditory detection and identification tasks designed to tax processing capacity. Tasks were constructed to highlight both the benefits and disadvantages of increased capacity. Autistics were better able to notice and respond to unexpected sounds, meaning, sounds not central to the current task or focus. The authors concluded that autistics were able to process a greater amount of auditory information at any given time compared to neurotypical individuals.

## 2 The paradox of detailed-focused and Gestalt-focused processing

We just reviewed the long-standing understanding of autistic cognition as detail-focused. This appears to contradict a theory that is increasingly popular among speech-language pathologists (SLP) called Gestalt language processing (GLP). GLP proposes that some individuals, including many autistic individuals, learn language by memorizing and using larger “chunks” of language (like phrases or sentences) before they fully understand the individual words within those chunks (25, 26). Because our goal is to chart out future research that could reconcile these two broad perspectives, we first review the historical background and current arguments for GLP, just as we did regarding evidence about autistics having weak central coherence.

### 2.1 The insight igniting Gestalt language fervor: echolalic utterances are communicative

Echolalia was the term to describe the repetition of phrases used in a meaningless way, such as repeating an advertising jingle. The innovation at the heart of GLP was that echolalic utterances have communicative intent. Prizant (26, 27) observed that the echolalic utterances produced by his autistic clients could be understood as meaningful for caregivers who understood the purpose and context of use, even if these utterances were idiosyncratic and not directly interpretable. As an example, one autistic youth repeated “Are you a good witch or a bad witch?” as a stand-in for greeting a new person. Prizant (26) proposed that the children were using the echoes to communicate, albeit idiosyncratically.

During the same era, language acquisition theorists established that using unanalyzed whole chunks to communicate is a strategy many children employ during early language development (25, 28, 29). As part of extensive research on typical children’s language acquisition, Nelson (28, 29) discussed holistic phrases such as *Iwandat*, which would be used during social interaction; she referred to this as an expressive style of language learning. Counterposed to this was the referential style, meaning using isolated words as labels to refer to objects.

Thirty years later, another SLP, Marge Blanc, took up Prizant’s insight that repeated phrases have communicative intent. Blanc (30) worked to develop and popularize the idea of Gestalt processing, describing it in a manner that SLPs could use to instruct parents to help autistic children communicate. This was done in a neuroaffirming way. Blanc did not try to “normalize” autistic children’s language acquisition, but to advise caregivers on treating Gestalt language as communicative, so that language could be a useful tool rather than an obstacle. Blanc (30) argued the autistics’ path to language learning is rooted in a “a form of thinking that includes a whole experience or situation—and suggest(s) that, for many GLPs, Gestalt language processing is an intrinsic part of the emotional experience in which the language was first used” (p. 1282, 31).

GLP proposes that autistic people latch onto a “whole experience or situation” because it is difficult for them to parse the individual components (in this case, words). As part of evaluating this claim, we note it stands in opposition to the long-standing claim, reviewed earlier, that autistics struggle to see the coherent whole because they are overly focused on the details.

## 3 Resolving the paradox

There are a number of possible reconciliations. We first note that the paradox is less severe than one might assume, because how autism is characterized has evolved since the decades when weak central coherence was proposed by Happé (2, 10, 32). Autistic individuals are now understood to employ both detail-oriented and big-picture processing, albeit in a slightly different manner from neurotypical individuals (33).

### 3.1 New ideas about bottom-up vs. top-down processing

In recent decades, local-global tasks have been heavily scrutinized by researchers (e.g., 34). A view has solidified that local processing bias is a cognitive style and a preference rather than a disability (35–37; although note that this was also proposed in 32). Evidence for this is that even when autistic individuals (including children) default to responding at the local level, they are able to report the global level when instructed to do so (16, 33, 38).

An idea frequently discussed in the autism community is that autistic people process details first and then use the details to construct a big-picture perspective (39). Numerous online articles, blogs and popular science books now summarize and explain the view that autistics can construct big-picture views, but these are the end-goal of processing, not the starting point (e.g., 40, 41). One autistic researcher noted that autistic people appear to be “details-before-the-concept” thinkers, while non-autistic people are “concept-before-the-details” thinkers (39).

Some autistic advocates note that learning approaches may differ from neurotypical norms. Aiello (42) advises: “If learning a scientific theory, an Autistic student may need to see multiple specific experiments and results before understanding the overarching concept, rather than absorbing a general explanation first.” Numerous social media posts elaborate on the view that autistics reach the big picture via processing of details, rather than being limited to details (43). However, this view is currently not discussed by academic researchers (as determined in July 2025 via multiple literature searches, including queries using AI tools).

This sea-change away from the original ‘weak central coherence’ view reduces the processing difference between weak central coherence and Gestalt processing in language. This raises the possibility that differences in the nature of tasks and one’s expertise could shift relative the extent to which someone makes use of detail-oriented vs. holistic processing.

### 3.2 No paradox, because echolalic utterances are not holistic episodic memories

GLP has been criticized from within the field of speech and language pathology (e.g., 44, 45) and from academic researchers (e.g., 46), for being ill-defined and lacking robust empirical support. Hutchins et al. (45) noted that both Prizant (26) and Peters (25) intended their description of Gestalt processing as provisional, awaiting future theoretical analysis. Yet Blanc (30) adopted and developed the idea of a holistic style as if it were a theoretically and empirically vetted construct. Some researchers have argued that clinical practices based on GLP could even be detrimental for children with neurodevelopmental and communication disabilities (47).

We focus here on the criticism of Hutchins et al. (45) that GLP is ill-defined because echolalic utterances are not unanalyzed forms, and are not holistic representations. Regarding forms not being analyzed, Hutchins et al. (45, p. 5) note that Prizant’s examples of “Gestalt mode of processing” appeared to be better characterized as “need for sameness but not a lack of internal analysis of the situation”.

Prizant (26) defined a Gestalt mode of processing as one “in which events are remembered or retained with relatively little analysis … [which] must be viewed in contrast to an analytic mode in which experiences or events are analyzed and segmented into meaningful components based upon prior experience” (p. 300). How can a phrase (which is a multi-word utterance) be remembered with relatively little analysis? Blanc and others proposed that Gestalt forms are supported by superior episodic memory, which allows information to exist in an unanalyzed, holistic form.

Hutchins et al. (45) claim this is a misunderstanding of episodic memory. Cognitive psychologists view episodic memory to be flexible, hierarchically structured, and composed of multiple analyzed components (48, 49). Episodic memory is a constructive process, not just a simple recording of past events. This constructive nature allows individuals to flexibly extract and recombine elements of past experiences, and to imagine or simulate future scenarios. Memories need to be reshaped slightly by new context, emotions, or suggestions during each retrieval. The reason for this is the human memory system is designed for predicting future events, which means it needs to be adaptable, not a mechanism for freezing in place replicas of past experiences (50).

Hutchins et al. (45) argued that delayed echolalia would be better understood as reflecting strength in perceptual memory. This is a non-declarative form of memory that registers and retains relatively unprocessed ‘snapshot’ records of single items to compensate for episodic memory (48, 51, 52).

Let us accept the point of Hutchins et al. (45) that echolalic utterances are recordings in perceptual memory. These could also be understood as holistic. It is thus difficult to understand why relatively unanalyzed forms stored as unprocessed snapshots in perceptual memory are not Gestalt forms. The proposal by Hutchins et al. (45) is thus either wrong or incomplete. Below, we propose several novel resolutions.

### 3.3 Echolalic utterances are details seized upon in a rich, complex conversational setting

If Gestalt forms are perceptual representations, they can plausibly exhibit enhanced sensory capacity. Consistent with existing research that autistics have enhanced auditory capacity, overheard phrases can be retained as an auditory trace. These can be holistic, in the sense that the phrase is mapped to some salient meaning in the environment, but not fully analyzed into constituent pieces.

Some phonological analysis must have been performed, since with echolalia, phonemes are typically pronounced correctly, and appropriate intonation is employed. But if autistics have local processing bias and attention to detail, why don’t they analyze the details of phrases, such as the component words? We propose that autistic learners may already be showing local processing bias regarding language input, but in a way that deviates from our conventional understanding of what it means to be detail-oriented in the domain of language.

Consider the richness and complexity of a typical conversational setting. Within this rich setting, a detail-oriented processor with high perceptual capacity may have selected the detail consisting of a phrase spoken to them or an overheard TV jingle. This is the detail that is selected for focus, but not integrated with diverse aspects of the larger setting in which the phrase is spoken, including objects handled by speakers and speakers’ physical actions in space. Consistent with local processing bias, the detail is not integrated within the larger conversational setting, which includes speakers’ communicative intentions. The result is one of the hallmarks of echolalia: unconventional, idiosyncratic meaning. Schuler and Prizant (53) cited the example from Kanner (54) of an autistic youth repeating *“don’t throw the dog off the balcony”* to remind himself to exert self-control. On this account, there is no conflict between GLP and detail-oriented processing, because an echolalic utterance is a detail, albeit also a holistic, unanalyzed form.

Given that autistics appear to excel at identifying the small units of any input stream, why would this ability be absent in the case of speech? The standard explanation is that identifying word-level semantics requires intense attention to joint attention and speakers’ communicative intent (55). Autistics at the beginning of language learning may be focusing their attention on perceptual details of the speech stream, rather than tracking conversation partners’ communicative goals.

A speculative proposal about why autistics sometimes do not break into language learning with words continues with the theme that autistics do have “enhanced auditory capacity.” Infants’ restricted working memory capacity was an assumption in Newport’s (56) ‘less is more’ hypothesis. Newport (56) speculated that early childhood is a sensitive period for language learning, due in part to reduced working memory capacity. According to the ‘less is more’ hypothesis, reduced capacity forces young children to focus on (and thus identify) smaller linguistic units that form the fundamental components of language (although see 57). In contrast, the more capacious working memory capacity of older children and adults allows them to encode large units of language, which then interferes with identifying what are the small units that form the building blocks of language.

### 3.4 Inherent differences between auditory and visual processing mean holistic processing is more easily observed in language than in vision

Auditory inputs can be easily repeated (re-produced) in a holistic fashion. This is as simple as listening to auditory input and repeating verbatim an overhead phrase or environmental sound. Any individual with a larger-than-expected sensory capacity can impress observers by repeating a long phrase. This can strike observers as ‘holistic’ if the speaker is not known to produce any of the phrases’ component words on their own.

Recall that visual stimuli where local processing bias is reported for autistic children and adults. What would it mean to demonstrate holistic processing in vision? An example is drawing. Autistics do produce high-capacity visual stimuli in the form of accurate, detailed drawings of real-life scenes (58). As example, is the autistic artist Stephen Wiltshire, who regularly draws pictures of cityscapes after a one-time viewing from a plane (59). This ability to produce high-capacity representations of visual scenes has been observed for autistic individuals beyond those labeled as savants. High-capacity drawings may be less apparent to observers because drawing requires fine motor skills, an area of documented challenges for some autistics (60). Nonetheless, when autistic children produce such drawings, they are lauded and admired, but what gets remarked by both caregiver and autism researchers is not the holistic nature of the drawings, but their details. We suggest that autistic visual processing is not just detail-oriented, but also has a holistic quality, given that some drawings are an accurate rendering of a coherent, big-picture stimulus, just as echolalic utterances are typically faithful copies of a heard phrase.

On this analysis, autistics’ enhanced sensory function allows them to repeat large sequences of auditory stimuli and re-present 2-D visual forms via drawing (or demonstrate knowledge via keen spatial navigation or rapid learning of visual material). We maintain that what differs between vision and audition is not that the former is detail-oriented and the latter holistic. What differs is that demonstrating to observers examples of holistic auditory forms is easily done through verbal repetition, but less easily done in the visual realm.

### 3.5 The paradox of weak-central coherence vs. Gestalt processing is an illusion caused by observers’ normative expectations, influenced by culture

When learning language, experts and parents alike have an expectation that words will be acquired first, then phrases. The building blocks should be acquired first, then combined into large forms. When autistics produce phrases, onlookers regard this as aberrant. The assumption that words must come first reflects a society that is educated about developmental psychology. This assumption is part of the educated middle-class parenting tool kit, not a universal of human language acquisition. Child language acquisition researchers have reported that typically developing children use multiple strategies in learning language, including phrasal productions (25, 28, 61).

In a different culture, the normative expectations may differ. For example, cross-cultural psychologists report that North Americans process a visual scene by focusing on a central detail. For example, when shown an image of a fish foregrounded in a fishtank, Americans report seeing “a fish”, while East Asians more often report the larger context, such as a fishtank (62). Stages of learning may also be different in cultures where children begin learning via overhearing rather than direct verbal interaction with caregivers (63).

The language domain has other examples where autistic children’s patterns are ambiguous between supporting weak central coherence vs. a lack of orientation to normative expectations. Autistic children do not manifest a shape bias in laboratory experiments on new word learning (64, 65). In contrast, the non-autistic group robustly used shape to infer the meaning of new words starting at 24 months of age, a phenomenon that exists (Hou et al. One interpretation is that learning to use shape to infer words’ meaning is an example of an abstraction of a high-level principle, and that its absence indicates weak central coherence (specifically, lack of generalization). An alternative is that autistic children have not learned (or not attended to) the normative expectation that shape is routinely used to identify words’ referents in typical language interactions. Agent-based modeling has been used to explain the shape bias as originating from pressures of communicative efficiency cultures (66). The strength of the shape-bias holds in some cross-cultural comparisons (e.g., 67), but not across others, suggesting that cultures vary in their normative expectations that object shape is relevant to inferring a word’s referent (68).

## 4 Discussion

Our purpose is to galvanize the research community to investigate whether autistics’ local processing bias and high-capacity productions in vision and languages share more similarities than is currently acknowledged. Visual outputs may have holistic properties (such as Wiltshire’s expert drawings), and echolalic utterances may have more details (such as precise phonemic and intonational renderings) than researchers have recognized. Here, we discuss the relevance of these issues to open questions in autism research.

### 4.1 Some autistics may be bottom-up processors, others, top-down processors

Across a range of types of cognitive processing, abilities are normally distributed for the population at large, but abilities for autistic individuals may manifest a more flattened distribution of abilities. This derives from more individuals at the low end of ability (corresponding to intellectual and social disabilities), but also a higher proportion of individuals with special skills, such as visual-spatial processing, music, and math ability (69, 70). A case where autistic abilities load more strongly on the tails of a distribution concerns sensory sensitivities. Some autistics are hypersensitive to sound and others are hyposensitive (71, 72). Hyposensitivity and hypersensitivity are not mutually exclusive but often co-occur within an individual (73, 74). The same individual may be hypersensitive to light but hyposensitive to sound. Individuals may even be hyper- and hyposensitive to the same sensory domain, with touch being the most commonly reported. We suggest the same variation can manifest in bottom-up vs. top-down processing.

But could some autistics be primarily top-down processors? The idea of autistic gestalt-type of cognition was suggested as far back as Kanner (75):

“[Autistic children’s] world must seem to them to be made up of elements that, once they have been experienced in a certain setting or sequence, cannot be tolerated in any other setting or sequence; nor can the setting or sequence be tolerated without all the original ingredients in the identical … order. (P.41)”.

We add here a contemporary updating. Autistic adults who have read about Gestalt language processing have reflected that they feel their cognitive style has Gestalt elements. Examples include the following.

I am a Gestalt processor, and this shows up in many different ways in my life. It is very hard for me to follow steps and thought processes unless I know the endpoint/the whole picture (77).

…when it comes to learning and thinking about things I have to first understand the greater context before focusing on specific pieces…. [subsequent response] I have always knew my brain or “way of thinking” was top down, iterative, and associative but never had the words for it past that (78).

### 4.2 Future research

Whether and how much some autistic individuals have a Gestalt approach vs a detailed orientation is unknown, with heterogeneous findings on global-local tasks (36). Future work can explore the relative strength of top-down vs. bottom-up processing across modalities, following what has been done by child language acquisition researchers (e.g., 61).

Some autistic individuals may:

- display a detail-focused approach to visual stimuli and a gestalt-focused approach to language and auditory processing.
- experience challenges when moving between details and gestalts, rather than being biased towards one or the other.
- have the ability to be detail-focused and holistic, with their major challenge located at an intermediary level of processing.

GLP is confined to language learning, whereas weak-central coherence is assumed to be part of general processing, although most examples concern vision. Future work could disentangle this difference when trying to reconcile whether or not there are different processing strategies across modalities, including and beyond vision vs. language.

### 4.3 Conclusions

The scientific research literature has characterized the autistic cognitive style as detail-oriented. As noted, this contrasts with the Gestalt language perspective discussed by speech-language pathologists. We described several ways to resolve this, such as understanding how large perceptual capacity can facilitate detailed, oriented processing in vision but holistic-seeming productions in speech. Autistic and neurotypical individuals differ in their connections between sensory processing and amodal integration systems (76). Future research can reveal how variations in connectivity may differently prioritize top-down vs. bottom-up information processing. Pursuing these questions between and within autistic and neurotypical groups will illuminate a core question presented in brain sciences, which is the nature of the relationship between low-level sensory information and higher-order cognition.
